# Molecular epidemiology of Japanese encephalitis in northern Vietnam, 1964–2011: genotype replacement

**DOI:** 10.1186/s12985-015-0278-4

**Published:** 2015-04-01

**Authors:** Loan Phuong Do, Trang Minh Bui, Futoshi Hasebe, Kouichi Morita, Nga Thi Phan

**Affiliations:** National Institute of Hygiene and Epidemiology, Hanoi, 10000 Vietnam; Institute of Tropical Medicine, Nagasaki University, Nagasaki, 852-8523 Japan

**Keywords:** Japanese encephalitis virus, JEV, Genotype replacement, E gene

## Abstract

**Background:**

*Japanese encephalitis virus* (JEV) is an arthropod-borne virus causing serious public health issues in Asia. JEV consists of five genotypes and recent studies have shown the emergence of JEV genotype I (GI) and its replacement of genotype III (GIII). Using an archival JEV collection, we investigated the molecular evolution of JEV in Vietnam over the last 48 years (1964–2012) in humans, mosquitoes, and pigs, within the global context.

**Methods:**

The nine JEV isolates from humans, pigs, and mosquitoes sequenced in this study and 29 sequences available in GenBank were used to analyze the envelope (E) protein of the Vietnamese JEVs. A collection of 225 cerebrospinal fluid specimens from patients with suspected Japanese encephalitis (JE) was also tested and genotyped with real-time RT–PCR.

**Results:**

The 38 E genes identified with sequencing and nine Vietnamese JEV strains genotyped with real-time RT–PCR, belonging to two lineages, evolved in accordance with those in the rest of the world. The first GIII strain was detected in humans in Vietnam in 1964, and in mosquitoes in 1979, whereas GI strains were first detected in humans and mosquitoes in 1990 and 1994, respectively. After 2004, GI was the only genotype detected in Vietnam, demonstrating that the GIIII strains had been displaced by GI strains. Five haplotypes were identified in the Vietnamese JEVs, with SKSS predominant. The S123N and S123R substitutions in the E protein were already present in the Vietnamese JEVs.

**Conclusion:**

This study describes the long evolutionary history of JEV in Vietnam over 34 years, which correlates well with the global evolution of JEV. The Vietnamese GIII strains have been replaced by GI strains in mosquitoes, pigs, and humans. The predominant haplotypes of the Vietnamese strains support this genotype displacement in Vietnam. Further surveillance is required to confirm the disappearance of the GIII strains in nature and the emergence of new pathogens causing encephalitis in Vietnam, after the long-term use of JEV vaccines in that country.

**Electronic supplementary material:**

The online version of this article (doi:10.1186/s12985-015-0278-4) contains supplementary material, which is available to authorized users.

## Background

*Japanese encephalitis virus* (JEV) is a mosquito-borne virus of the genus *Flavivirus* in the family *Flaviviridae*. Although the clinical symptoms caused by JEV were first described in Japan in 1897, the prototype Nakayama strain was only detected in patient with encephalitis in 1935 in Japan [1,2]. Since its first report in humans, JEV has circulated widely in Asian and western Pacific countries, and in northern Australia, and an estimated 3 billion people live in the endemic region. Approximately 67,900 cases of JE typically occur annually and 10,000–15,000 JEV-related human deaths are reported annually [2,3].

The JEV genome consists of a single-stranded positive-sense RNA, approximately 11 kb in length. The open reading frame (ORF) encodes a large polyprotein that is cleaved into at least 10 proteins. The N-terminal region of the polyprotein encodes the structural proteins (C–prM–E), followed by the nonstructural proteins (NS1–NS2A–NS2B–NS3–NS4–NS5) [4].

JEV is transmitted to susceptible reservoirs by arthropods. The natural cycle of JEV is maintained with the involvement of mosquitoes, especially *Culex tritaeniorhynchus*. Pigs, horses, birds, and bats are the primary natural hosts of JEV, and humans are the dead-end host.

Based on the nucleotide sequences of the C/prM and envelope (E) protein genes, JEV is classified into five genotypes and the cut-off value for the nucleotide differences between each genotype is 12% [5]. Although all five genotypes have been detected in mosquitoes and reservoirs, only genotypes I and III are predominant in humans. There is only one JEV genotype II strain in Australia, one strain in Korea around 1951, and one JEV genotype V strain in Malaysia in 1952 [5-7]. Genotype I (GI) consists of two clades, GI-a and GI-b, and the majority of strains belong to GI-b. Genotype I strains have displaced genotype III (GIII) strains to become the predominant genotype in many countries [8]. The emergence of JEV GI strains was first identified in mosquitoes and pigs in northern Asian countries, Korea in 1993 [9] and Japan in 1994 [10,11], and in mosquitoes in China in 1979 [12]. A recent phylogeographic analysis indicated that GI-a strains circulate in Thailand and Cambodia, whereas GI-b strains were emerged in Vietnam, dispersing northwards into China, and then into Japan, Korea, and Taiwan. However, these GI strains have mainly been isolated from mosquitoes and swine, and very few have been isolated from humans. Therefore, the JEV GI strains are considered to be more adapted to mosquitoes and pigs than to humans [13]. Twelve haplotypes are defined based on the four sites in the E protein (sites 123, 209, 227, and 408) that are predicted to be under positive selection. The predominant haplotypes are SKSS and SKPS [14]. The haplotypes and the host ranges of the GI isolates are narrower than those of the GIII isolates.

In Vietnam, the first JEV strains were isolated in 1960 in humans, pigs, and birds. Before 1990, all the JEV strains isolated were GIII [15]. In 2001–2002, a second genotype, GI, was first reported in mosquito and swine isolates from northern Vietnam [13]. Taking advantage of an archival collection of JEV strains isolated in Vietnam between 1964 and 2011, we conducted this study to examine the molecular epidemiology of the JEV strains circulating in humans, mosquitoes, and pigs in Vietnam in 1964–2011, specifically placing our observations within the global context.

## Results

### Emergence of GI JEVs in Vietnam and their replacement of GIII

**Figure 1:**
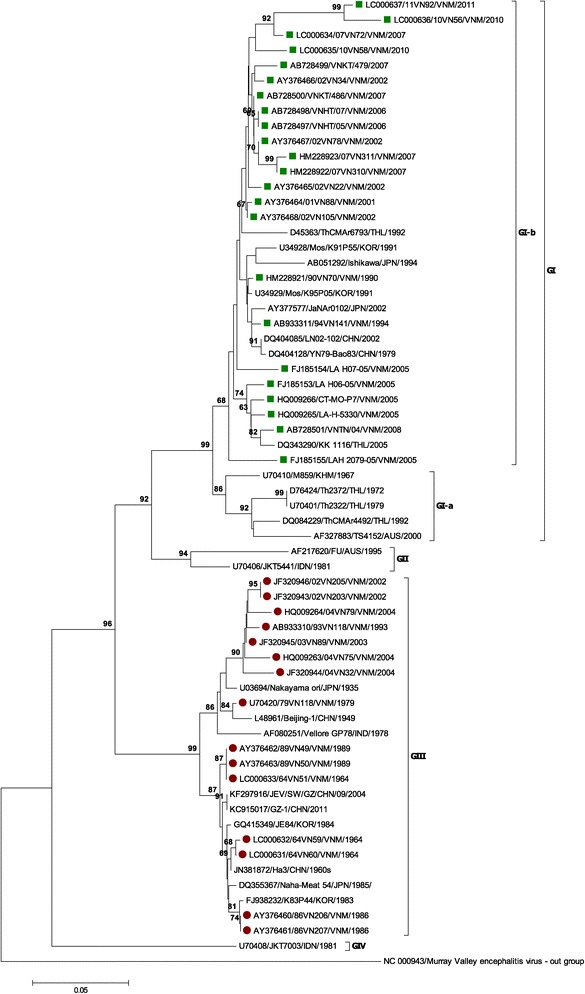
**Phylogenetic tree showed the relationship between Vietnamese JEV and others.** The tree was constructed on the envelope (E) nucleotide sequences of 38 Vietnamese JEV strains and other reference strains. GI and GIII sequences obtained in this study are marked in red circle and blue square, respectively. Genotypes and sub-gentoypes are indicated on the right-side. Scale bar indicates nucleotide substitutions per site.

A phylogenetic tree was constructed using the 38 Vietnamese E gene nucleotide sequences isolated in Vietnam in 1964–2011, together with 25 E gene sequences obtained from the DNA databases of other countries (Figure 1). Of the 38 Vietnamese JEV strains, 17 isolates were from mosquitoes, six were from swine, and 15 were from patients with confirmed JE. The Vietnamese JEV strains clustered into two genotypes, GI and GIII. All the Vietnamese GI strains belonged to the GI-b clade defined by Schuh et al. which includes the major GI strains circulating worldwide, including in China, Japan, Korea, and Thailand, whereas subgenotype GI-a contains strains circulating only in Thailand and Australia [8]. All the JEVs isolated from mosquitoes and pigs before 1994 were genotype GIII, including the prototype Nakayama and Beijing-1 strains. After 1994, all the JEV strains isolated from mosquitoes and pigs were genotype GI. In humans, before the first detection of a GI strain in 1990, all the isolates were genotype GIII (Additional file 1: Table S1). After the first GI strain was reported in humans, it was 17 years before the second JEV GI strain was detected in humans in 2007. During the period from 1990 to 2004, JEV GIII strains were continuously isolated from humans in northern Vietnam, and no Vietnamese isolates belonged to GII, GIV, or GV (Figure 1).

We used the highly sensitive real-time RT–PCR technique and genotype-specific probes and primers to detect and genotype the JEVs in the CSF specimens from patients with suspected JE in the National Hospital of Pediatrics, Hanoi, Vietnam, from 2008 to 2012. We collected 45 CSF specimens in June every year, the peak of the JE season in northern Vietnam. The average positive rate detected with real-time RT–PCR was 4% (9/225), and no specimen was positive in 2009 or 2010. However, the average positive rate was 23.1% (4.4–42.2%) when an IgM antibody capture ELISA was used for the diagnosis of JE. The highest JE-positive rates were 11.1% and 42.0% in 2008 when real-time RT–PCR and ELISA were used, respectively. Three specimens were positive on both ELISA and real-time RT–PCR. Therefore, 25.78% of the 225 samples from patients with suspected JE were positive when both detection methods were used (ELISA and real-time RT–PCR) (Additional file 2: Table S2).

All nine positive CSF specimens confirmed with real-time RT–PCR were genotype GI, but six of them could not be detected with IgM antibody directed against JEV (data not shown).

The nucleotide identity among the Vietnamese GIII strains was 96.7%. The first Vietnamese GIII strain (64VN51) and the prototype Nakayama strain shared 96.4% nucleotide identity and the 64VN51 and Beijing-1 strains shared 95.6%. The identity between the Vietnamese GIII strains and one Chinese strain (GZ/CHN/09) was 85.9%.

The nucleotide identity between the Vietnamese GI strains was 96.9%. The nucleotide identity between the first Vietnamese GI JEV strain (90VN70) and other strains in clade GI-b was 97.1%, whereas that between 90VN70 and strains in clade GI-a was 93.1%.

### Molecular characteristics of the E protein in Vietnamese JEV strains from 1964 to 2011 at sites under positive selection

**Figure 2:**
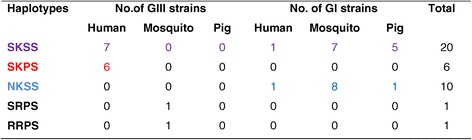
**GI and GIII haplotypes for E protein.**

Five JEV haplotypes are detected in the isolates from humans, pigs, and mosquitoes in Vietnam (Figure 2): four halotypes in GIII strains (SKSS, SKPS, SRPS, and RRPS), but only two haplotypes (SKSS and NKSS) in GI strains. The haplotype SKSS was found in both the GI and GIII strains. However, SKPS was only found in the GIII strains and NKSS was only found in the GI strains, and were the second most common haplotypes in each genotype. Haplotypes RRPS and SRPS were found in one GI strain each, in mosquito isolates. The haplotypes were more divergent in the mosquito isolates, which displayed all five haplotypes, than in the human or pig isolates, in which only two haplotypes were identified.

## Discussion

A molecular epidemiological study of the E genes of Vietnamese JEV strains isolated over the last 47 years (1964–2011) showed the gradual replacement of GIII by GI. The GIII strains became less dominant when the GI strains emerged and became predominant. In mosquitoes and pigs, genotype displacement occurred after 1994, whereas in humans, the GI and GIII strains co-circulated between 1990 and 2004. However, since 2004, only GI strains have been detected in the human population. To determine the circulation of the GIII strains in nature during the period from 2005 to 2007, we attempted to isolate JEVs from 300 CSF specimens collected from patients in the acute phase of suspected JE during the peak epidemic season, but only one GI JEV strain was isolated, in 2007. Therefore, we used highly sensitive real-time RT–PCR with specific primer sets for the GI and GIII strains to analyze the genomic material of JEV strains isolated from 225 CSF samples collected in the peak JE seasons (June) between 2008 and 2012. However, we isolated only six GI strains and no GIII strain from this series of samples.

The most reliable information used in molecular epidemiological surveillance is based on the nucleotide sequence of the E/PrM gene in the isolated JEV strains. However, the rate of successful isolation of JEV from human CSF specimens is very small because the human immune response is very rapid. JEVs are also very sensitive to temperature, so it is difficult to isolate the virus from CSF specimens and then detect the JEV genomic material with conventional RT–PCR. Therefore, highly sensitive real-time RT–PCR is used to detect JEV in humans, and when genotype-specific primer sets are used, the genotypes of the JEV strains in specimens can be identified [16]. The epidemic season for JE is summer in subtropical countries, such as northern Vietnam, peaking in June. In this study, we collected CSF samples from JE-suspected patients admitted to the National Hospital of Pediatrics, Hanoi, in June, at the peak of the JE season [17] and analyzed them with real-time RT–PCR and genotype-specific primer sets for GI and GIII [18], but found no GIII strains in the CSF specimens. This result demonstrates the continuous displacement of GIII by GI strains. Notably, although real-time RT–PCR is highly sensitive, the average positive rate of infection determined with an ELISA that detects IgM antibodies directed against JEV was 23.1%, much higher than the rate (4%) when real-time RT–PCR was used with the same specimen collection. This comparison strongly confirms why the detection of specific antibodies directed against JEV in CSF samples or in paired sera with an IgM capture antibody ELISA is the gold standard technique for JEV diagnosis. However, no antibodies were detected in six of the nine CSF samples that tested positive with real-time RT–PCR, because the antibody titer is low in the acute phase of the disease.

Many recent studies have shown that GI strains have emerged and displaced GIII strains to become the predominant genotype throughout Asian countries, including Korea, China, India, Taiwan, and Japan [8,11-13,19,20]. In Australia, although a GII strain was the first genotype detected in 1995 [5], after only 5 years, GI strains had emerged as the second most common genotype in 2000 [21]. However, in these countries, although GI strains are predominant, GIII strains are still cocirculating with GI strains. In China, GIII strains were still found in pigs in central China in 2010 [22]. In Thailand, although only GI strains were isolated, that study was limited to the mosquito population. The same scenarios have also been reported in Korea [23], Malaysia [24], and India [20], where only GI strains were detected, although only in limited mosquito populations.

We have presented evidence of the close relationships between the Vietnamese JEV GIII strains and other strains from northern Asia, based on the nucleotide sequence of an archival collection of JEV GIII strains collected in different regions of Vietnam (northern, central, southern, and highland) in 1964–2011. The nucleotide identity between the first JEV GIII strain isolated in Vietnam in 1964 and the prototype Nakayama strain (Japan, 1935, 97.1%) was higher than that between it and Beijing-1 (China, 1959, 95.6%) or a Chinese strain (85.9%). When the nucleotide sequences of the E gene of the JEV GI strains in Vietnam were analyzed, the strains most similar to the first Vietnamese GI strain were Korean strain K95P05, Japanese strain JaNAr0102, and Chinese strain YN79-Bao83, with identities of 97.1%, whereas the identity between the first Vietnamese GI strain and Thai, Cambodian, and Australian GI strains was 93.1%. This prompted the hypothesis that the GI-b strains emerged in Vietnam, dispersed northward to China and then to Japan and Taiwan, whereas the GI-a strains circulated in a confined area in Thailand and Cambodia, from where they spread to Australia [8]. Although clade GI-b contains some Thai strains, those strains are thought to circulate locally [25].

As this study demonstrates, after the emergence of JEV GI in the 1990s, it was 10 years before the JEV GIII strains became “quiescent” in 2004. Despite a huge effort to identify GIII strains circulating in nature, we found no GIII strains after 2004. We infer that the GIII strains have totally disappeared or are still maintained at very low levels in nature that are undetectable with current surveillance methods. The cycle of emergence and “quiescence” of a pathogen has been recorded previously, including the “quiescent time” of the *O’nyong’nyong virus* responsible for fever in Africa from 1959 to 1996 [26]. A similar genotype shift was also identified in *Dengue virus* (DENV), a mosquito-transmitted virus of the genus *Flavivirus*. One of the most characteristic patterns of DENV evolution is that the genotypes emerge and disappear gradually. DENV serotype 2 in Vietnam, which belongs to the Asian 1 lineage, was probably introduced into southern Vietnam in the late 1990s and subsequently displaced the Asian/American lineage of serotype 2 viruses [27].

The relationship between the JEV GI and GIII haplotypes of the E protein and genotype displacement is noteworthy. In both the Vietnamese GI and GIII strains, the predominant haplotype is SKSS in humans, pigs, and mosquitoes. The SKPS haplotype contains an amino acid substitution of serine with proline at residue 227 in the E protein, which might be related to the retarded growth of this haplotype in Vero cells [14,28]. This may partly explain why this haplotype was restricted to GIII strains in humans in this study, and to GIII strains in humans and mosquitoes worldwide, rather than to the currently predominant GI strains. The mutation at this site might affect viral virulence associated with membrane fusion [29]. The NKSS haplotype, containing an asparagine at position 123, was the predominant haplotype detected in GI strains in mosquitoes, and is the predominant genotype worldwide. To our knowledge, only one strain containing asparagine has been isolated from humans in Thailand, in 1985. In this study, we identified one more isolate from humans which contains the S123N substitution. The RRPS haplotype, which contains an S123R substitution (in the E protein), increases the growth rate of JEV, and alters its pathogenicity in mice [28], was already present in one Vietnamese GIII strain in mosquitoes in 1979.

Notably, almost 75% of the 225 CSF specimens from patients with encephalitis were negative for JEV. This result might be related to the recent emergence of a novel virus in Vietnam [30]. Close surveillance is required to identify the encephalitic pathogens and the “quiescent” GIII strains in the future.

## Conclusion

In this study, we provide strong evidence for the complete displacement of JEV genotype GIII by genotype GI based on the E gene sequence of 38 JEVs isolated in Vietnam in 1964–2011 and the JEV genotypes in 225 CSF specimens collected from patients with suspected JE in 2008–2012. The evolution of the Vietnamese JEV strains correlates well with that of global JEVs. Further studies are required to monitor both the “quiescent” GIII strains and the emerging novel pathogens that cause encephalitis in Vietnam.

## Methods

### Ethical statement

This study was approved by the Institutional Review Board of the National Institute of Hygiene and Epidemiology, Vietnam (no. 08 IRB, June 11, 2012).

### Specimen collection

In total, 225 cerebral spinal fluid (CSF) specimens were collected from patients with suspected JE, who were admitted to the National Hospital of Pediatrics in Hanoi, Vietnam in June each year from 2008 to 2012. All CSF specimens were collected in the first 5 days after disease onset (acute phase) and stored at –80°C until analysis with real-time RT–PCR.

The nine JEV strains sequenced in this study were from the archival CSF brain tissues collected from patients with acute encephalitis syndrome, or from mosquito pools in northern Vietnam between 1964 and 2011. The samples were inoculated into C6/36 cells. Two days after inoculation, the cell culture fluid was harvested for RNA extraction.

### RNA extraction, real-time RT–PCR, and conventional RT–PCR

The genomic RNAs were extracted from the infected cell culture fluids and CSF specimens with the QIAamp Viral RNA Mini Kit (Qiagen Sciences, Germantown, MD, USA), according to the manufacturer’s instructions.

To detect and differentiate GI and GIII directly in the CSF specimens, the total RNA was amplified with the SuperScript® III Platinum® One-Step qRT–PCR Kit (Life Technologies, Carlsbad, CA, USA) and genotype-specific primer sets for GI (JE-E1-2140 F 5′- GGGGACAAGCAGATTAACCA-3′, JE-E1-2325R 5′-GAAGGCACCACCAAACACTT-3′, and JE-E1-2200Probe 6-FAM-TCAACAACTTTGAAAGGGGC-TAMRA) and GIII (JE-E3-1978 F 5′- CCTTGCAAAATTCCGATTGT -3′, JE-E3-2222R 5′-TGAGCTCCCTTCAAAGTCGT - 3′, and JE- E3-2038Probe: 6-FAM-CTGGTGACAGTGAACCCCTT-TAMRA) [18].

To amplify the E gene of JEV for sequencing and the phylogenetic analysis of nine JEV strains, conventional RT–PCR was performed with the Qiagen OneStep RT–PCR Kit (Qiagen GmbH, Hilden, Germany), with a primer set specific for the E gene [13].

### Immunoglobulin M (IgM) capture enzyme-linked immunosorbent assay (ELISA) to detect JEV

An IgM capture ELISA was used to detect IgM anti-JEV antibodies in CSF specimens from patients suspected of clinical JE, using the commercial kit *JE MAC*-*ELISA* (National Institute of Hygiene and Epidemiology, Hanoi, Vietnam).

### Sequencing and phylogenetic analysis

After purification with the QIAquick PCR Purification Kit (Qiagen), the PCR products were sequenced with the BigDye Terminator Cycle Sequencing Ready Reaction Kit, version 3.1 (Applied Biosystems, Foster City, CA, USA). The nucleotide sequences were determined on an ABI Prism 3130 Genetic Analyzer (Applied Biosystems). The contigs were aligned with the SeqMan program in the Lasergene 8 software package (DNASTAR, Inc. Madison, WI, USA).

We compared the E gene nucleotide sequences determined in this study with those of Vietnamese strains available in GenBank (38 E gene nucleotide sequences detected in Vietnam, including the nine JEV strains sequenced in this study). Twenty-five JEV strains from GenBank were used as the reference strains in the analysis (Additional file 1: Table S1). *Murray Valley encephalitis virus* was chosen as the outgroup for the phylogenetic tree.

The sequences were aligned with ClustalW [31] and the phylogenetic analysis was performed with the MEGA package version 6.0 [32]. To construct the phylogenetic tree, the neighbor-joining method and the distance method were used [33]. The distance topology was compared with the maximum likelihood method to identify any differences. The statistical significance of the phylogenetic tree was estimated with a bootstrap analysis with 1,000 pseudoreplicate datasets. We used the complete deletion option to eliminate positions containing missing data.

#### Nucleotide sequence accession numbers

The sequences (n = 9) of the E genes identified in this study were submitted to the DDJB/GenBank/EMBL databases under accession numbers: [DDBJ: LC000631– DDBJ: LC000637] and [DDBJ: AB933310 and DDBJ: AB933311].

### Consent

Written informed consent was obtained from the patient’ guardian/parent/next of kin for the publication of this report and any accompanying images.

## Additional files

Additional file 1: Table S1. Information of JEV strains used in this study*. Additional file 2: Table S2. Detection of JE cases among JE clinical suspected cases by real-time RT-PCR and IgM antibody capture ELISA, 2008 – 2012.

## Abbreviations

JEV: Japanese encephalitis virus
CSF: Cerebrospinal fluid
RT–PCR: Reverse transcription–polymerase chain reaction
GI: Genotype I
GIII: Genotype IIII
ELISA: Enzyme-linked immunosorbent assay
IgM: Immunoglobulin M
