# Supplementary material for: Molecular epidemiology of Japanese encephalitis in northern Vietnam, 1964–2011: genotype replacement
Source: Virol J. 2015 Apr 1;12:51. doi: 10.1186/s12985-015-0278-4 (PMC4417254; doi:10.1186/s12985-015-0278-4)
Supplement: Additional file 1: Table S1. — Information of JEV strains used in this study*. [file 12985_2015_278_MOESM1_ESM.doc]

Supplementary Table 1. Information of JEV strains used in this study*

| **Order** | **Strain** | **Year of collection** | **Country** | **Host** | **Genotype** | **Accession No.** |
| --- | --- | --- | --- | --- | --- | --- |
|  | **64VN51** | **1964** | **Vietnam** | **Human brain** | **G III** | **LC000630** |
|  | **64VN59** | **1964** | **Vietnam** | **Human brain** | **G III** | **LC000632** |
|  | **64VN60** | **1964** | **Vietnam** | **Human brain** | **G III** | **LC000631** |
|  | 79VN118 | 1979 | Vietnam | Mosquito | G III | U70420 |
|  | 86VN206 | 1986 | Vietnam | Human brain | G III | AY376460 |
|  | 86VN207 | 1986 | Vietnam | Human brain | G III | AY376461 |
|  | 89VN49 | 1989 | Vietnam | Human brain | G III | AY376462 |
|  | 89VN50 | 1989 | Vietnam | Human brain | G III | AY376463 |
|  | 90VN70 | 1990 | Vietnam | Blood of JE case | G I | HM228921 |
|  | **93VN118** | **1993** | **Vietnam** | **Mosquito** | **G III** | **AB933310** |
|  | **94VN141** | **1994** | **Vietnam** | **Mosquito** | **G I** | **AB933311** |
|  | 01VN88 | 2001 | Vietnam | Swine | G I | AY376464 |
|  | 02VN22 | 2002 | Vietnam | Swine | G I | AY376465 |
|  | 02VN34 | 2002 | Vietnam | Mosquito | G I | AY376466 |
|  | 02VN78 | 2002 | Vietnam | Mosquito | G I | AY376467 |
|  | 02VN105 | 2002 | Vietnam | Mosquito | G I | AY376468 |
|  | 02VN203 | 2002 | Vietnam | CSF of JE case | G III | JF320943 |
|  | 02VN205 | 2002 | Vietnam | CSF of JE case | G III | JF320946 |
|  | 03VN89 | 2003 | Vietnam | CSF of JE case | G III | JF320945 |
|  | 04VN32 | 2004 | Vietnam | CSF of JE case | G III | JF320944 |
|  | 04VN75 | 2003 | Vietnam | CSF of JE case | G III | HQ009263 |
|  | 04VN79 | 2004 | Vietnam | CSF of JE case | G III | HQ009264 |
|  | LA-H-06-05 | 2005 | Vietnam | Swine | G I | FJ185153 |
|  | LA-H07-05 | 2005 | Vietnam | Swine | G I | FJ185154 |
|  | LAH_2079-05 | 2005 | Vietnam | Swine | G I | FJ185155 |
|  | LA-H-5330 | 2005 | Vietnam | Swine | G I | HQ009265 |
|  | CT-Mo-P7 | 2005 | Vietnam | Mosquito | G I | HQ009266 |
|  | VNHT/05/2006 | 2006 | Vietnam | Mosquito | G I | AB728497 |
|  | VNHT/07/2006 | 2006 | Vietnam | Mosquito | G I | AB728498 |
|  | VNKT/479/2007 | 2007 | Vietnam | Mosquito | G I | AB728499 |
|  | VNKT/486/2007 | 2007 | Vietnam | Mosquito | G I | AB728500 |
|  | 07VN 310 | 2007 | Vietnam | Mosquito | G I | HM228922 |
|  | 07VN 311 | 2007 | Vietnam | Mosquito | G I | HM228923 |
|  | VNKT/04/2008 | 2008 | Vietnam | Mosquito | G I | AB728501 |
|  | **07VN 72** | **2007** | **Vietnam** | **CSF of JE case** | **G I** | **LC000634** |
|  | **10VN 56** | **2010** | **Vietnam** | **Mosquito** | **G I** | **LC000636** |
|  | **10VN 58** | **2010** | **Vietnam** | **Mosquito** | **G I** | **LC000635** |
|  | **11VN 92** | **2011** | **Vietnam** | **Mosquito** | **G I** | **LC000637** |
|  | FU | 1995 | Australia | Human blood | G II | AF217620 |
|  | JKT5441 | 1981 | Indonesia | Mosquito | G II | U70406 |
|  | TS4152 | 2000 | Australia | Mosquito | G I | AF318290 |
|  | ThCMAr6793 | 1963 | Thailand | Mosquito | G I | D45363 |
|  | Th2372 | 1972 | Thailand | Human brain | G I | D76424 |
|  | Th2322 | 1979 | Thailand | Human brain | G I | U70401 |
|  | ThCMAr4492 | 1992 | Thailand | Mosquito | G I | DQ084229 |
|  | JE_KK_1116 | 2005 | Thailand | Swine | G I | DQ343290 |
|  | M859 | 1967 | Cambodia | Mosquito | G I | U70410 |
|  | GP78 | 1978 | India | Human brain | G III | AF080251 |
|  | JKT7003 | 1981 | Indonesia | Mosquito | G IV | U70408 |
|  | K83P44 | 1983 | Korea | Mosquito | GI | FJ938232 |
|  | JE84 | 1987 | Korea | Mosquito | GIII | GQ415349 |
|  | K91P55 | 1991 | Korea | Mosquito | G I | U34928 |
|  | K95P05 | 1994 | Korea | Mosquito | G I | U34929 |
|  | Beijing-1 | 1949 | China | Human brain | G III | L48961 |
|  | H3a | 1960s | China | CSF of JE case | GIII | JN381872 |
|  | YN79-Bao83 | 1979 | China | Mosquito | G I | DQ404128 |
|  | LN02-102 | 2002 | China | Mosquito | G I | DQ404085 |
|  | GZ09 | 2004 | China | Swine | GIII | KZ297916 |
|  | GZ-1 | 2011 | China | Swine | GIII | KC915017 |
|  | Nakayama original | 1935 | Japan | Human brain | G III | U03694 |
|  | Naha-Meat54 | 1985 | Japan | Swine | GIII | DQ355367 |
|  | Ishikawa | 1994 | Japan | Swine | G I | AB051292 |
|  | JaNAr0102 | 2002 | Japan | Mosquito | G I | AY377577 |

*The strains in bold were sequenced in this study.
