# Supplementary material for: Molecular epidemiology of Japanese encephalitis in northern Vietnam, 1964–2011: genotype replacement
Source: Virol J. 2015 Apr 1;12:51. doi: 10.1186/s12985-015-0278-4 (PMC4417254; doi:10.1186/s12985-015-0278-4)
Supplement: Additional file 2: Table S2. — Detection of JE cases among JE clinical suspected cases by real-time RT-PCR and IgM antibody capture ELISA, 2008 – 2012. [file 12985_2015_278_MOESM2_ESM.doc]

**Supplementary Table 2. Detection of JE cases among JE clinical suspected cases by real-time RT-PCR and IgM antibody capture ELISA, 2008 – 2012**

| **Year** | **IgM antibody capture ELISA** | | **Real-time RT-PCR** | |
| --- | --- | --- | --- | --- |
|  | **Tested specimen** | **Positive (%)** | **Tested specimen** | **Positive (%)** |
| 2008 | 45 | 19 (42.2) | 45 | 5 (11.1) |
| 2009 | 45 | 2 (4.4) | 45 | 0 (0.0) |
| 2010 | 45 | 13 (28.8) | 45 | 0 (0.0) |
| 2011 | 45 | 4 (8.8) | 45 | 2 (4.4) |
| 2012 | 45 | 14 (31.1) | 45 | 2 (4.4) |
| **Total** | **225** | **52 (23.11%)** | **225** | **09 (4%)** |
